# Peripheral blood leukocyte signatures as biomarkers in relapsed ovarian cancer patients receiving combined anti‐CD73/anti‐PD‐L1 immunotherapy in arm A of the NSGO‐OV‐UMB1/ENGOT‐OV30 trial

**DOI:** 10.1002/1878-0261.13811

**Published:** 2025-01-30

**Authors:** Luka Tandaric, Annika Auranen, Katrin Kleinmanns, René DePont Christensen, Liv Cecilie Vestrheim Thomsen, Cara Ellen Wogsland, Emmet McCormack, Johanna Mäenpää, Kristine Madsen, Karen Stampe Petersson, Mansoor Raza Mirza, Line Bjørge

**Affiliations:** ^1^ Centre for Cancer Biomarkers CCBIO, Department of Clinical Science University of Bergen Norway; ^2^ Department of Obstetrics and Gynecology Haukeland University Hospital Bergen Norway; ^3^ Department of Obstetrics and Gynecology and Tays Cancer Centre Tampere University Hospital Finland; ^4^ Nordic Society of Gynaecological Oncology – Clinical Trial Unit (NSGO‐CTU) Tampere Finland; ^5^ Nordic Society of Gynaecological Oncology – Clinical Trial Unit (NSGO‐CTU) Copenhagen Denmark; ^6^ Department of Health Registry Research and Development Norwegian Institute of Public Health Oslo Norway; ^7^ Kinn Therapeutics AS Bergen Norway; ^8^ Centre for Pharmacy, Department of Clinical Science University of Bergen Norway; ^9^ Department of Internal Medicine, Hematology Section Haukeland University Hospital Bergen Norway; ^10^ Faculty of Medicine and Health Technology Tampere University Finland; ^11^ Department of Oncology Rigshospitalet, Copenhagen University Hospital Denmark

**Keywords:** biomarkers, durvalumab, immunotherapy, liquid biopsy, oleclumab, ovarian cancer

## Abstract

Immune checkpoint inhibitors have demonstrated limited efficacy in overcoming immunosuppression in patients with epithelial ovarian cancer (EOC). Although certain patients experience long‐term treatment benefit, reliable biomarkers for responder pre‐selection and the distinction of dominant immunosuppressive mechanisms have yet to be identified. Here, we used a 40‐marker suspension mass cytometry panel to comprehensively phenotype peripheral blood leukocytes sampled over time from patients with relapsed EOC who underwent combination oleclumab (anti‐CD73) and durvalumab (anti‐PD‐L1) immunotherapy in the NSGO‐OV‐UMB1/ENGOT‐OV30 trial. We found that survival duration was impacted by baseline abundances of total peripheral blood mononuclear cells. Longitudinal analyses revealed a significant increase in CD14^+^CD16^−^ myeloid cells during treatment, with significant expansion of monocytic myeloid‐derived suppressor cells occurring in patients with shorter progression‐free survival, who additionally showed a continuous decrease in central memory T‐cell abundances. All patients demonstrated significant PD‐L1 upregulation over time on most T‐cell subsets. Higher CD73 and IDO1 expression on certain leukocytes at baseline significantly positively correlated with longer progression‐free survival. Overall, our study proposes potential biomarkers for EOC immunotherapy personalization and response monitoring; however, further validation in larger studies is needed.

## Graphical abstract

Using mass cytometry, we analyzed serial blood samples from patients with relapsed epithelial ovarian cancer (EOC) treated with oleclumab–durvalumab combination immunotherapy in the NSGO‐OV‐UMB1/ENGOT‐OV30 trial. Our analysis identified potential predictive, monitoring, and response biomarkers detectable through liquid biopsy. These findings facilitate the advancement of strategies for patient pre‐selection and treatment personalization to address the limited efficacy of immunotherapy in EOC.

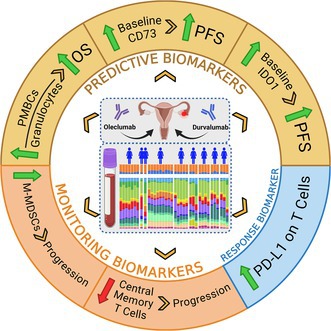

**Abbreviations**
CSB: cell staining buffer
CyTOF: cytometry by time‐of‐flight
ECOG: Eastern Cooperative Oncology Group
EOC: epithelial ovarian cancer
FIGO: International Federation of Gynecology and Obstetrics
ICI: immune checkpoint inhibitor
M‐MDSC: monocytic myeloid‐derived suppressor cell
*P*_adj_
: adjusted *P*‐value
PARPi: poly(ADP‐ribose) polymerase inhibitor
PBMC: peripheral blood mononuclear cell
PBS: phosphate‐buffered saline
PFS: progression‐free survival
RT: room temperature
T_CM_
: central memory T cell
UMAP: Uniform Manifold Approximation and Projection

## 1 Introduction

Epithelial ovarian cancer (EOC) is predominantly diagnosed at an advanced stage. For the past few decades, surgical cytoreduction followed by carboplatin–paclitaxel chemotherapy has been the default approach to treating EOC [1]. Recently, the clinical implementation of poly(ADP‐ribose) polymerase inhibitors (PARPis) and the anti‐angiogenesis agent bevacizumab as maintenance therapy has significantly improved survival [2]. However, despite most patients initially responding to primary treatment, 70–80% of the responders eventually experience relapse [3]. Although multiple therapeutics are used to treat recurrent disease, the treatment intent shifts from curative to palliative. Drug resistance and the dearth of effective post‐relapse treatment options have resulted in long‐term survival rates remaining low [4] and have highlighted the need for novel treatment modalities.

Immunotherapy, particularly the clinical implementation of immune checkpoint inhibitors (ICIs), has improved the outcomes of several cancers by disrupting tumor immune evasion mechanisms, mainly the induction of T‐cell exhaustion [5]. Seminal studies by Zhang et al. [6] and Curiel et al. [7], correlating treatment response and survival with the presence, position, and phenotypes of tumor‐infiltrating lymphocytes in EOC, have indicated rationale for applying immunotherapy in EOC as well. Although numerous trials on immunotherapeutic regimens in EOC have been conducted, including studies incorporating patient pre‐selection strategies based on predictive biomarkers, such as PD‐L1 [8], none have shown sufficient efficacy to be considered clinically viable [9]. Immunotherapy has failed to achieve potency in EOC on account of multiple immunoinhibitory mechanisms simultaneously active in its tumor microenvironment. This facilitates a high degree of immunosuppression, unsurmountable by immunotherapeutics delivered either as a monotherapy or in combination with chemotherapeutics [10]. In preclinical studies on EOC, combination immunotherapy regimens have resulted in notable increases in antitumor efficacy over single‐agent immunotherapeutics, predominantly by inhibition of PD‐1/PD‐L1 interaction alongside simultaneous activation of an immunostimulatory receptor on T cells [11].

Building off of promising preclinical results of combination immunotherapies in murine models of solid cancers, which had shown significant synergistic effects in terms of tumor reduction and survival improvement, as well as elevated abundance and anti‐tumor activity of CD8^+^ T cells, compared to the corresponding monotherapies [12, 13, 14], the non‐randomized phase II trial NSGO‐OV‐UMB1/ENGOT‐OV30 was designed as a three‐arm umbrella trial for testing the clinical efficacy of several novel combinations of immunotherapeutics in patients with relapsed EOC [15]. The treatment in arm A comprised oleclumab and durvalumab ‐ human monoclonal IgG1 antibodies that target and inhibit CD73 and PD‐L1, respectively. CD73, an adenosine‐generating extracellular enzyme present in most normal tissues [16], is overexpressed in most cancers [17] and associated with poor prognosis in EOC [18]. Binding of adenosine to A2A receptors on antigen‐activated T cells results in activation blockage and exhaustion [19]. Accordingly, inhibition of the CD73/A2A receptor axis is considered an effective means of reinvigorating anti‐tumor immunity [20]. Furthermore, simultaneous inhibition of CD73 and the PD‐1/PD‐L1 interaction in preclinical *in vitro* and *in vivo* human cell line models was shown to have a synergistic anti‐tumor effect compared to the individual inhibition of either [21]. Based on these results, the oleclumab/durvalumab combination was approved for testing in a phase II clinical trial setting [15]. While it demonstrated marginal clinical efficacy in a relapsed EOC cohort, resulting in a median progression‐free survival of 2.7 months, a median overall survival of 8.4 months, and a disease control rate of 27%, with only one patient showing a response, a number of patients nonetheless exhibited prolonged survival.

This translational study presents the results of the high‐dimensional single‐cell suspension cytometry by time‐of‐flight (CyTOF) analysis of blood samples collected from trial cohort A prior to treatment and at regular intervals during treatment. The aim was the elucidation of clinically actionable phenotypic signatures of the response of relapsed EOC to oleclumab/durvalumab immunotherapy.

## 2 Materials and methods

### 2.1 Patient cohort and samples

**Fig. 1:**
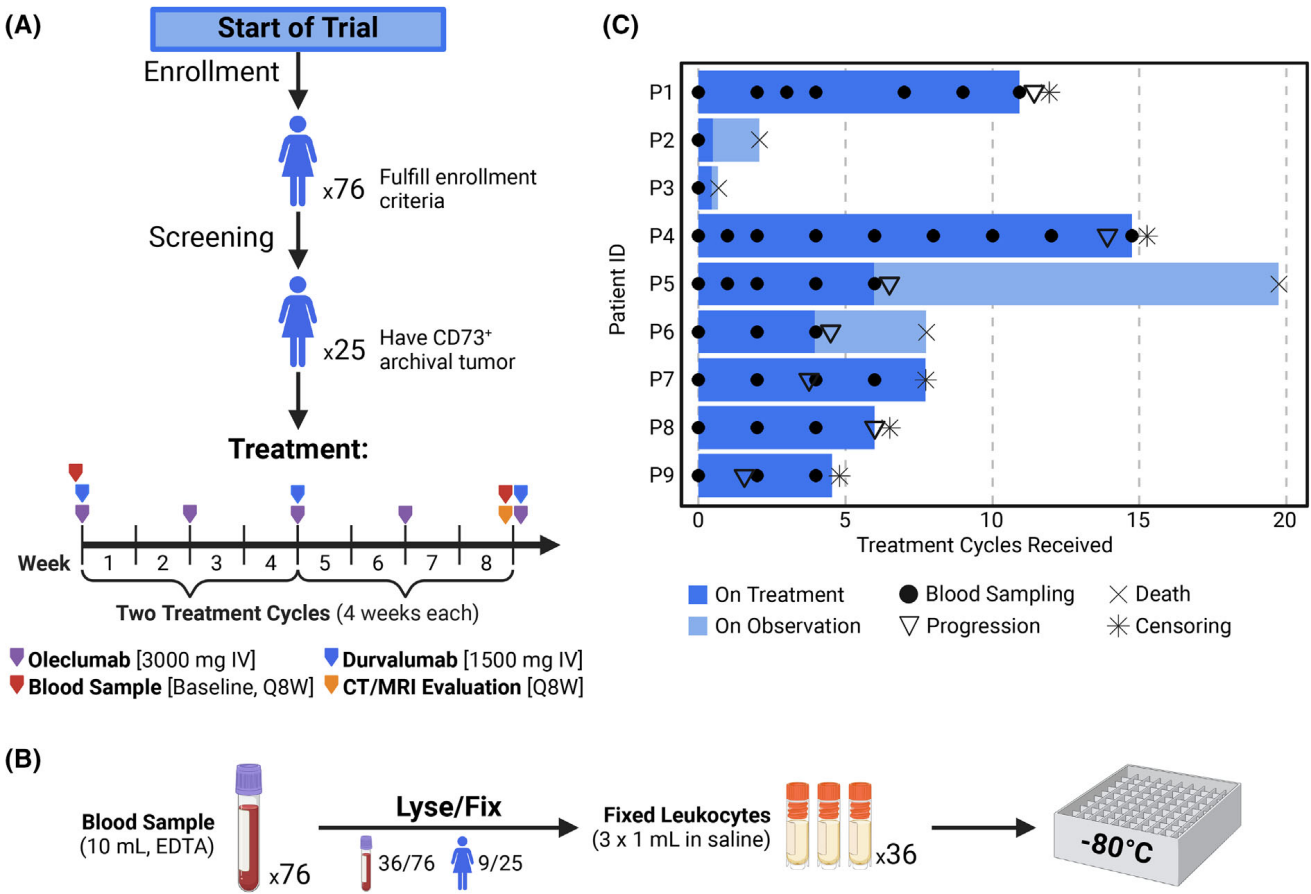
Trial overview, sample collection, and processing. (A) Schema of the patient disposition and immunotherapy administration schedule of the NSGO‐OV‐UMB1/ENGOT‐OV30 trial. One treatment cycle had a duration of 4 weeks. (B) Blood sample acquisition and processing workflow (step‐by‐step protocol available as Supporting Information). (C) Swimmer plot illustrating the duration of treatment and observation for each patient included in this study (*n* = 9), as well as blood sampling timepoints. IV, intravenous; Q8W, every 8 weeks.

In the international NSGO‐OV‐UMB1/ENGOT‐OV‐30 umbrella trial, 25 immunotherapy‐naïve patients with relapsed EOC who had CD73‐positive archival tumor samples were included in arm A and intravenously administered 3000 mg oleclumab and 1500 mg durvalumab every 2 and 4 weeks, respectively (Fig. 1A). The primary endpoint was response evaluation (disease control rate) at the 16‐week timepoint. Peripheral blood samples for single‐cell suspension CyTOF were collected in 10 mL EDTA Vacutainer tubes (Becton Dickinson, Franklin Lakes, NJ, USA, REF 367525) at baseline (pre‐treatment), at minimum every two 28‐day cycles, and at the end of the trial or at disease progression. Within 1 h of collection, samples were processed using BD Phosflow Lyse/Fix buffer (Becton Dickinson, REF 558049) and frozen at −80 °C (Fig. 1B,C) (step‐by‐step protocol available as Data S1). Samples were stored in the biobank of Haukeland University Hospital, Bergen, Norway. The study, including its protocol and the use of patient material, was approved by the regional ethics committees or institutional review boards of the participating sites: The Scientific Ethics Committee for the Capital Region of Denmark (VEK) (approval no. H‐17025483), The Regional Committee for Medical Research Ethics Western Norway (REK West) (approval no. 2018/580), and The Regional Ethics Committee of the Expert Responsibility area of Tampere University Hospital (TAYS) (approval no. R18078M). The study was conducted in accordance with the good clinical practice guidelines and provisions of the Declaration of Helsinki, as well as according to all local regulations. All patients provided written informed consent for the use of the clinical information and biological materials.

### 2.2 Mass cytometry panel development

A 40‐marker antibody panel (Table S1) encompassing the entire human peripheral blood leukocyte repertoire was designed and developed in‐house for in‐depth single‐cell analysis of the leukocyte samples. As the aim of oleclumab/durvalumab immunotherapy was the reinvigoration of T cells from an exhausted state, a central objective of the panel was ascertainment of information about T‐cell states before and during treatment. Given that myeloid cells in the peripheral blood have been observed to significantly influence the outcome of immunotherapy of solid cancers [22], as well as have a crucial influence on treatment outcome upon settlement into the tumor microenvironment of EOC [23], another objective of the panel was the characterization of the myeloid cells in the samples.

For antibody clones not commercially available in a pre‐metal‐conjugated format, 100 μg of purified, carrier‐protein‐free antibody was conjugated in‐house to cadmium‐ or lanthanide‐loaded polymers using Maxpar MCP9 or X8 antibody labeling kits, respectively, according to the manufacturer's protocol (Standard BioTools, South San Francisco, CA, USA).

Antibodies were titrated on leukocytes from whole blood collected from healthy female donors and processed using the Lyse/Fix buffer. Titration of lineage and cell state markers was performed on unstimulated leukocytes, and peripheral blood mononuclear cells (PBMCs) stimulated *in vitro* by interleukin‐2 (Cat. no. I2644; Sigma, St. Louis, MI, USA) and phytohemagglutinin‐P (Cat. no. 61764; Sigma), respectively (step‐by‐step protocol available as Supporting Information). To ensure consistency, identical sample processing and staining protocols were maintained for the titration samples and clinical samples. The optimal concentration of Cell‐ID Intercalator‐Ir (Cat. no. 201192B; Standard BioTools) for the identification of nucleated cells was determined to be 250 nM by titration. The panel was validated for use on the CyTOF XT mass cytometer (Standard BioTools).

### 2.3 Sample staining and data acquisition

To facilitate uniform staining, batches containing up to 20 samples were designed, wherein a unique palladium‐based tag was assigned to each sample. For batch effect correction, each barcoded batch contained two different anchor samples consisting of Lyse/Fix‐treated healthy donor leukocytes, one of which had an admixture of phytohemagglutinin‐P‐/interleukin‐2‐treated healthy donor PBMCs to enable batch correction of activation/exhaustion markers. Frozen fixed leukocyte samples were thawed and treated with 0.25 mg·mL^−1^ DNAse I (Cat. no. DN25; Sigma) dissolved in Dulbecco's phosphate‐buffered saline containing Ca^2+^ and Mg^2+^ (Cat. no. D8662; Sigma) (henceforth referred to as “DNAse”). Cells were counted, and a maximum of 3.5 × 10^6^ cells from each sample were tagged using a palladium‐based barcoding method according to the manufacturer's protocol (Cat. no. 201060; Standard BioTools). Barcoding reagents were washed away, and all cells within a batch were pooled, counted, and frozen at −80 °C.

To maintain consistency in the composition of staining antibody mixes across sample batches, two mixes of antibodies, targeting either surface or intracellular markers, were pre‐made in quantities large enough to stain the full set of samples, aliquoted, and stored at −80°C. Comparative testing of frozen versus freshly made antibody mixes on Lyse/Fix‐treated healthy donor leukocytes demonstrated that freezing did not affect the effectiveness or specificity of the panel antibodies.

For staining, barcoded sample mixes were thawed and counted. Cells were then incubated in MaxPar Cell Staining Buffer (CSB) (Cat. no. 201068; Standard BioTools) containing human FcR Blocking Reagent (Cat. no. 130‐059‐901; Miltenyi Biotec, Bergisch Gladbach, Germany), and heparin (Reg. no. 5394.00.00; Ratiopharm, Ulm, Germany) to prevent non‐specific antibody binding. Aliquots of the antibody mix for staining cell surface proteins were thawed and added to the cells at 3 × 10^6^ cells per 100 μL of staining volume. After incubating for 45 min at room temperature (RT), superfluous antibody was washed away with MaxPar CSB, then with MaxPar phosphate‐buffered saline (PBS) (Cat. no. 201058; Standard BioTools), after which cells were permeabilized by a 10‐min incubation in pure methanol (Cat. no. 32213; Sigma) at −20°C. The methanol was washed away with MaxPar PBS, followed by MaxPar CSB. Aliquots of the antibody mix for targeting intracellular proteins were thawed and applied to the permeabilized cells for 30 min at RT, at a concentration of 3 × 10^6^ cells per 100 μL of staining volume. For DNA staining, washed cells were incubated with iridium intercalator diluted in MaxPar PBS containing 4% V/V formaldehyde for 20 min at RT. The cells were washed, resuspended in a 10% V/V solution of dimethyl‐sulfoxide (Cat. no. W387520; Sigma) in fetal bovine serum (Cat. no. F7524; Sigma), and frozen at −80°C.

For data acquisition, fully stained cells were thawed, washed using MaxPar CSB, and incubated in DNAse. Cells were then washed and pelleted in MaxPar Cell Acquisition Solution (Cat. no 201240; Standard BioTools), kept on ice until data acquisition, and acquired in MaxPar Cell Acquisition Solution Plus (Cat. no. 201244; Standard BioTools). High‐dimensional data from three barcoded batches were acquired on a CyTOF XT mass cytometer at ~ 400 events per second, with EQ Six‐Element Calibration Beads (Cat. no. 201245; Standard BioTools) present in the suspension at a 1 : 10 dilution (step‐by‐step barcoding and staining protocols are available as Supporting Information).

### 2.4 Data pre‐processing

Bead‐based longitudinal signal intensity normalization was done automatically by the cytof xt software upon data acquisition. Events were de‐barcoded using the matlab Compiler Runtime (R2013a(8.1)) implementation of The Single Cell Debarcoder by Zunder et al. [24]. De‐barcoded events were uploaded to Cytobank (Beckman Coulter Inc., Brea, CA, USA), where irregular events were removed by manual gating (Fig. S1). The resulting single‐cell events were batch corrected with the R‐based GUI implementation of CytofBatchAdjust by Schuyler et al. (v0.0.0.9001) [25], using the composite anchor sample of each batch. Batch correction effectiveness was assessed by analyzing the corrected data of the alternate anchor sample.

### 2.5 Clustering and differential analyses

Unsupervised clustering, dimensionality reduction, manually curated metaclustering, and differential analyses were performed using the “flowSOM” [26], “umap” [27], “CATALYST” [28], and “diffcyt” [29] R packages, respectively.

**Fig. 2:**
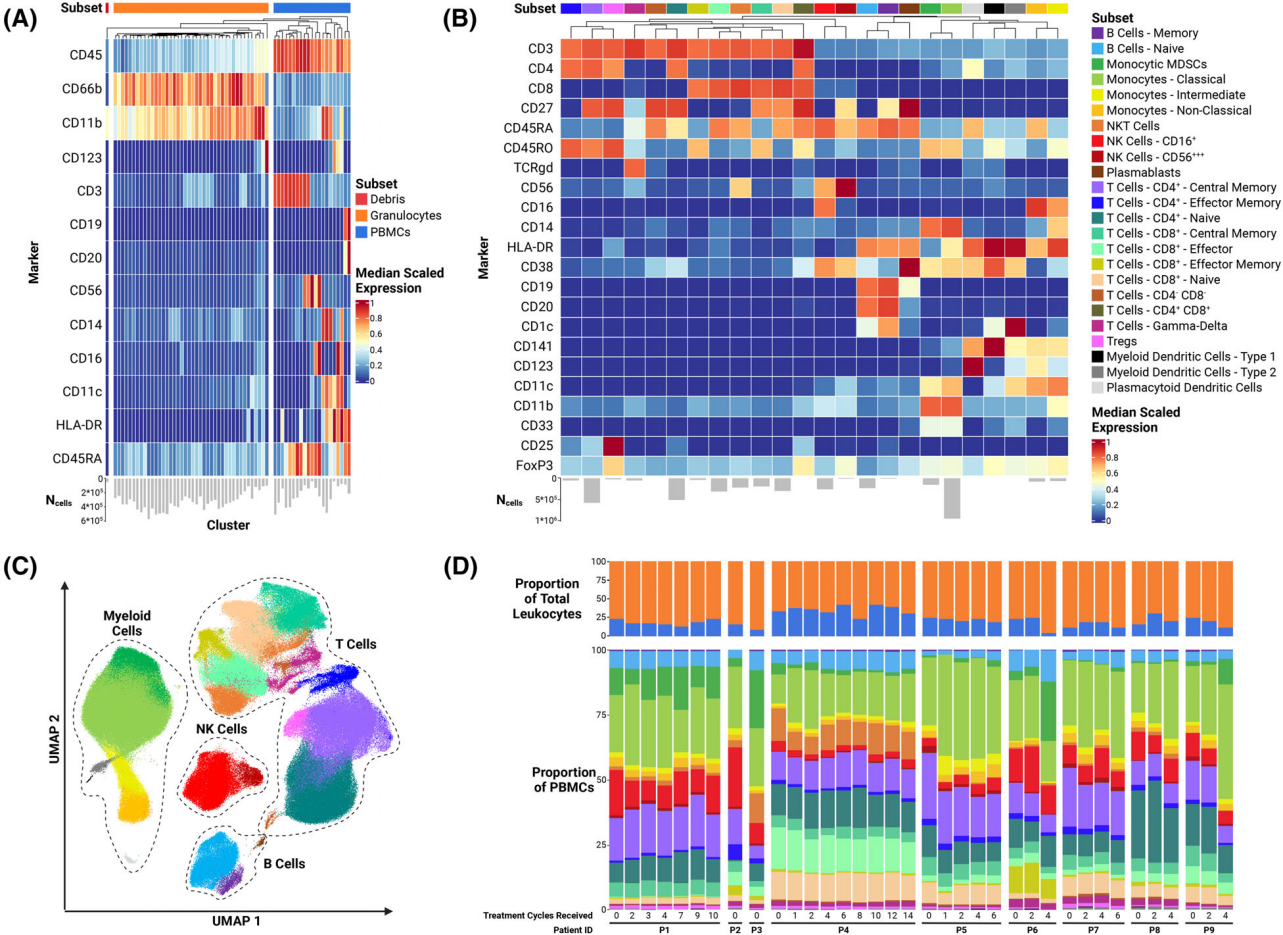
Formation and overall view of the study dataset. (A) Heatmap of the normalized median expression of the 13 markers (rows) used for the clustering of total leukocytes. Each column represents a cluster. Appropriateness of leukocyte subset assignment* was confirmed by unsupervised hierarchical clustering (visualized above heatmap). (B) Heatmap of the normalized median expression of the 22 markers (rows) used for the clustering of PBMCs. Each column represents a leukocyte subset. Subset assignment* was performed and validated in the same manner as for the total leukocyte clusters. (C) UMAP of an aggregated sample of PBMCs, consisting of an equal number of cells sampled from each blood sample. Each cell is colored by subset assignment*. (D) Overview of the leukocyte (upper stacked bar plots) and PBMC (lower stacked bar plots) composition of all blood samples (*n* = 36) (the detailed leukocyte composition of each sample is presented in Table S2). *Leukocyte subset assignment coloring is consistent throughout this figure. PBMCs, peripheral blood mononuclear cells; TCRgd, gamma‐delta T‐cell receptor; UMAP, Uniform Manifold Approximation and Projection.

Total leukocyte data from all patient samples were first clustered into a 64‐cluster (8 × 8) self‐organizing map and manually metaclustered into granulocyte, PBMC, and debris subsets based on the expression of 13 markers (Fig. 2A). The assignment of cell identity was rigorously quality checked using biaxial plots, heatmaps, UMAPs, and hierarchical clustering of clusters. The PBMC subset was further subclustered into a 256‐cluster (16 × 16) self‐organizing map and manually grouped into 24 subsets corresponding to widely established PBMC types using 22 markers (Fig. 2B). Subset assignment was validated in the same manner as that for total leukocytes.

### 2.6 Statistical analyses

Differential subset abundance and cell state marker expression between clinical sample groups, stratified by length of survival, duration of progression‐free survival (PFS), or sampling timepoint, were analyzed using the edgeR and limma functions of the diffcyt R package, respectively. Multiple hypothesis testing correction was conducted using the Benjamini–Hochberg method, giving adjusted *P*‐values (*P*_adj_).

To identify potential predictive biomarkers among baseline leukocyte characteristics, the relationships between PFS duration, and either relative cell subset abundances or state marker expression medians for each cell subset were analyzed using GraphPad Prism (v10.2.0; GraphPad Software, La Jolla, CA, USA). Given that patients in the short‐term survivor group had died prior to the first tumor evaluation timepoint, this analysis was restricted to the seven patients in the long‐term survivor group. According to the results of Shapiro–Wilk normality testing (alpha = 0.05), either a Pearson or Spearman correlation test was performed for each marker‐subset combination (dataset) alongside simple linear regression to assess the relationship between PFS duration and relative abundances or expression medians. The *P*‐values in the correlation analyses were not corrected for multiple hypothesis testing due to the small size of datasets (*n* = 7) and the discovery‐driven nature of this study. To maintain robustness, we considered significant (*P* < 0.05) correlations with an absolute correlation coefficient of at least 0.5 and an *R*‐squared value of at least 0.75 as meaningful.

## 3 Results

### 3.1 Study cohort demographics

In this translational study, we analyzed total leukocytes in prospectively collected longitudinal blood samples from patients with relapsed EOC undergoing combination oleclumab/durvalumab immunotherapy (Fig. 1A) with the aim of identifying predictive, prognostic, and response biomarkers for the administered treatment. Out of the 25‐patient trial cohort, 16 patients were not eligible for this study due to samples either missing or being of inadequate quality (Fig. 1B). Therefore, the data in this study stem from 36 leukocyte samples of nine patients (Fig. 1C). Nonetheless, our study cohort is highly representative of the clinical trial cohort [15], as the patient age range and distributions of International Federation of Gynecology and Obstetrics (FIGO) stage, Eastern Cooperative Oncology Group (ECOG) performance status, and clinical response are similar (Table 1). In contrast to the patients in the clinical trial, the majority of the patients included in this study had undergone more extensive treatment prior to inclusion.

**Table 1:** Characteristics of the included patients (n = 9). CR, complete response; ECOG, Eastern Cooperative Oncology Group; FIGO, International Federation of Gynecology and Obstetrics; PD, progressive disease; PR, partial response; SD, stable disease.

Characteristics	No. of patients (*%*)
Age, years	
Median (range)	66 (47–74)
40–49	1 (11.1)
50–59	1 (11.1)
60–69	3 (33.3)
≥ 70	4 (44.4)
FIGO stage at initial diagnosis	
I	1 (11.1)
II	0 (0.0)
III	3 (33.3)
IV‐A	4 (44.4)
IV‐B	1 (11.1)
ECOG performance status at baseline	
0	3 (33.3)
1	6 (66.7)
Prior treatment lines	
2	3 (33.3)
≥ 3	6 (66.7)
Treatment cycles received^a^	
Median (range)	6 (1–15)
≤ 4	4 (44.4)
> 4	5 (55.6)
Time to progression (weeks)	
≤ 16	3 (33.3)
> 16	4 (44.4)
N/A^b^	2 (22.2)
Best clinical response	
CR	0 (0.0)
PR	1 (11.1)
SD	5 (55.6)
PD	1 (11.1)
N/A^b^	2 (22.2)

^a^ One cycle corresponds to 4 weeks of treatment.

^b^ Patients passed away prior to the first post‐baseline tumor evaluation.

### 3.2 CyTOF analysis enabled in‐depth characterization of patients' leukocyte profiles

We utilized single‐cell suspension CyTOF for high‐dimensional phenotypic profiling of patient leukocytes. Approximately 21 million total events were acquired from the 36 leukocyte samples. After quality controls, single‐cell data of about 19 million leukocytes remained for further analysis. After batch correction, unsupervised clustering using FlowSOM and manual metaclustering of the total leukocytes produced three leukocyte subsets ‐granulocytes, PBMCs, and debris (Fig. 2A). To achieve the intended analytical depth, the same clustering and metaclustering procedures were applied in higher resolution to the PBMCs, resulting in the clear delineation of 24 PBMC subsets (Fig. 2B). The robustness of the PBMC (meta)clustering was confirmed by using the UMAP dimensionality reduction algorithm on an aggregated dataset comprising 10 000 PBMCs from each of the 36 samples (Fig. 2C).

All PBMC subtypes, except for the rarest populations (plasmablasts, CD141^+^ myeloid dendritic cells, and CD4^+^CD8^+^ T cells), were detected in all samples (Fig. 2D, Table S2). Inspection of the complete dataset revealed that each patient's leukocyte profile remained largely consistent over time, but inter‐patient heterogeneity was markedly more pronounced (Fig. 2D). Nevertheless, certain trends in compositional changes of leukocyte profiles over time were observed, such as the expansion of myeloid cells at the cost of overall T‐cell proportions in nearly all patients. To determine the significance of these observations for biomarker discovery, we performed differential analyses of leukocyte composition and cell state marker expression.

### 3.3 Long‐term survivors exhibit larger abundances of PBMCs at baseline

**Fig. 3:**
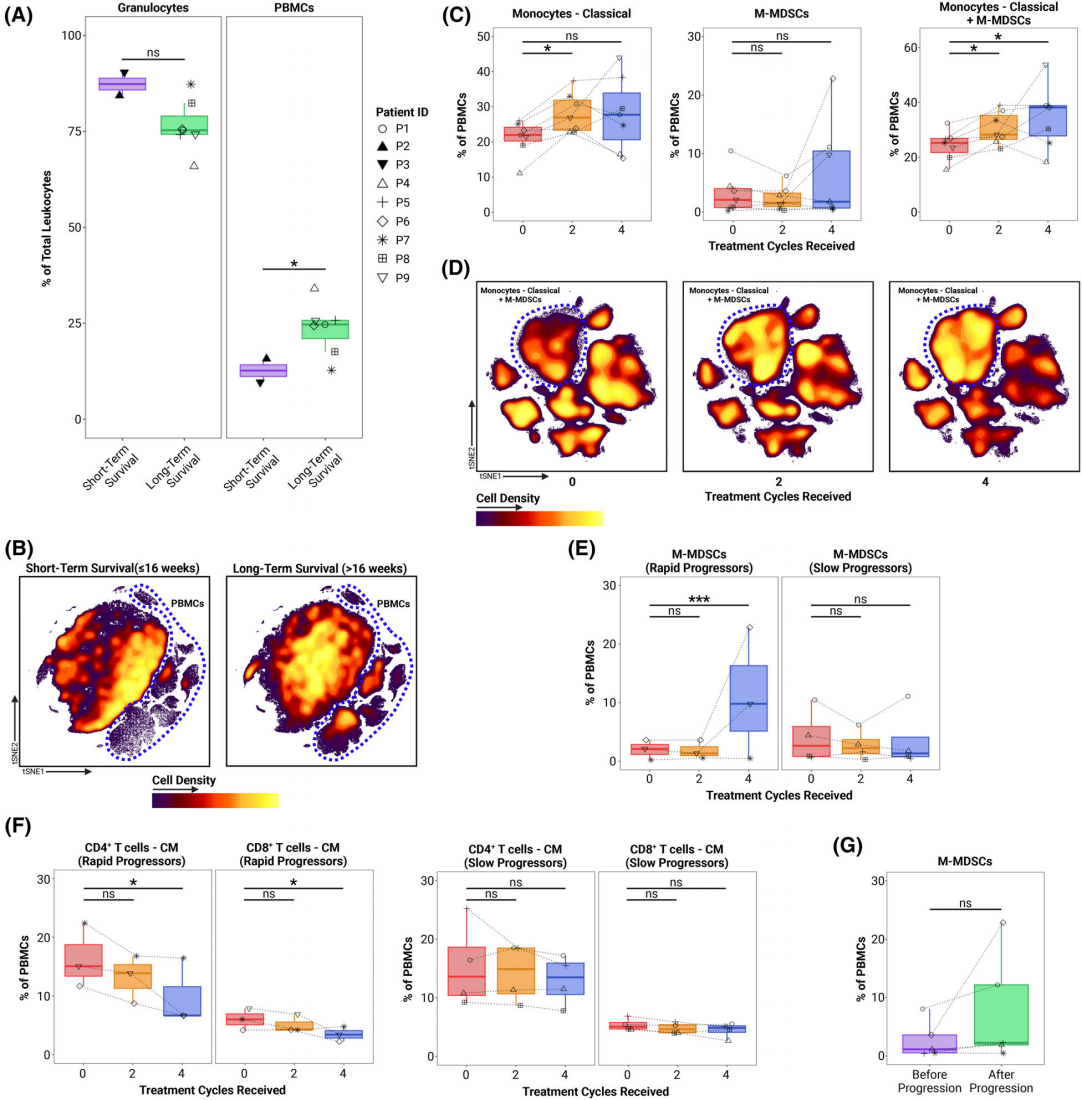
Significant differences, changes, and trends in the relative abundances of leukocyte populations. (A) Comparison of the relative baseline abundances of granulocytes and total PBMCs between survival duration groups (short‐term survivors (*n* = 2) were defined as patients demonstrating survival of at most 16 weeks since the start of treatment; long‐term survivors (*n* = 7) were defined as patients demonstrating survival of more than 16 weeks since the start of treatment). Symbols representing each patient are consistent throughout all figures. (B) UMAPs of aggregated baseline samples of total leukocytes stratified by survival duration group. Both projections consist of the same number of cells. The significant difference in the density of the total PBMC subset between groups is accented with a dashed blue outline. (C) Changes in the relative abundances of classical monocytes and M‐MDSCs during treatment in the group of long‐term survivors (*n* = 7). The two PBMC subsets composed of CD14^+^CD16^−^ myeloid cells (classical monocytes and M‐MDSCs) are shown separately in the left and middle panels. The level of HLA‐DR expression forms the basis for the separation of classical monocytes (HLA‐DR^+/hi^) from M‐MDSCs (HLA‐DR^−/lo^) (Fig. S3). In the right panel, the classical monocyte and M‐MDSC subsets were merged. (D) UMAPs of aggregated PBMC samples from the long‐term survivors (*n* = 7), stratified by treatment timepoint. Cells of the “Monocytes‐Classical” and “M‐MDSCs” subsets are delineated with a blue outline. (E) Changes in the relative abundances of M‐MDSCs during treatment. Patients were stratified into rapid progressors (disease progression confirmed after at most 16 weeks since the start of treatment) (*n* = 3) and slow progressors (disease progression confirmed after more than 16 weeks since the start of treatment) (*n* = 4). (F) Changes in the relative abundances of central memory T‐cell subsets during treatment. Patients were stratified into rapid (*n* = 3) and slow (*n* = 4) progressors. (G) Comparison of the relative abundances of M‐MDSCs in paired samples collected at blood sampling timepoints immediately before (*n* = 5) and after confirmation of disease progression (*n* = 5). Box and whisker plots represent the median (central line), interquartile range (IQR) (box), and minimum and maximum values within 1.5 × IQR out from the IQR (whiskers). *P*‐values were calculated using the “EdgeR” method of the diffcyt R package, which utilizes an overdispersed Poisson model, an empirical Bayes procedure, and an exact test adapted for overdispersed data [29], then corrected for false discovery rate using the Benjamini–Hochberg method. Statistical analyses involving multiple timepoints were performed on paired data. ns, not significant; *P_adj_ < 0.05; ***P_adj_ < 0.001. CM, central memory; MDSCs, monocytic myeloid‐derived suppressor cells; PBMCs, peripheral blood mononuclear cells; UMAP, Uniform Manifold Approximation and Projection.

To identify clinically relevant predictive biomarkers, patients were first stratified into short‐term survivors (≤ 16 weeks, *n* = 2) and long‐term survivors (> 16 weeks, *n* = 7) (Fig. S2). We observed that long‐term survivors had, on average, a significantly higher relative abundance of total PBMCs at baseline (*P*_adj_ = 0.0279) (Fig. 3A,B). No significant differences were observed in the relative abundances of other cell subsets between the groups at baseline (Table S3). As the short‐term survivors provided only baseline samples, we could not compare other timepoints between the groups.

### 3.4 Oleclumab/durvalumab treatment was accompanied by significant CD14^+^CD16^−^ myeloid cell expansion

To ensure consistency and sufficient sample size for adequate statistical power when analyzing longitudinal changes, we evaluated only the samples from long‐term survivors taken at baseline (*n* = 7) and after two (*n* = 7) and four treatment cycles (*n* = 7). Compared to baseline, the average frequency of classical monocytes (CD14^+^CD16^−^HLA‐DR^+/hi^) in the peripheral blood samples significantly increased (*P*_adj_ = 0.0248) after two treatment cycles and remained elevated even after four treatment cycles, although not significantly (*P*_adj_ = 0.299) (Fig. 3C, left panel). Although there were no significant differences in the relative abundances of monocytic myeloid‐derived suppressor cells (M‐MDSCs) (CD14^+^CD16^−^HLA‐DR^−/lo^) over time when analyzed separately from classical monocytes (Fig. 3C, middle panel), removing the division between classical monocytes and M‐MDSCs based on HLA‐DR expression (Fig. S3) revealed a significant expansion of CD14^+^CD16^−^ myeloid cells relative to baseline. This expansion was evident after two treatment cycles (*P*_adj_ = 0.0165) and persisted even after four treatment cycles (*P*_adj_ = 0.0231) (Fig. 3C, right panel). We visualized this progressive increase in abundance by using UMAP to generate density‐colored projections of aggregate samples of PBMCs from each timepoint (Fig. 3D).

### 3.5 Myeloid MDSC expansion and central memory T‐cell contraction are tied to progression onset

All seven patients in the long‐term survivor group experienced tumor progression and were subsequently categorized either as rapid progressors (*n* = 3), who experienced disease progression within the first 16 weeks (four cycles) of treatment, or slow progressors (*n* = 4), who had disease progression more than 16 weeks from the start of treatment (Fig. S2). Differential abundance analyses showed significant expansion of the M‐MDSC subset after four treatment cycles in the rapid progressors (*P*_adj_ = 6.89 × 10^−8^) (Fig. 3E, left panel), with no significant longitudinal changes in M‐MDSC abundance occurring in the slow progressors (Fig. 3E, right panel). Furthermore, among rapid progressors, we observed a consistent decline, leading to a statistically significant contraction, of both CD4^+^ (*P*_adj_ = 0.0477) and CD8^+^ (*P*_adj_ = 0.0477) central memory (CD27^+^CD45RO^+^CD45RA^−^) T‐cell (T_CM_) subsets after four treatment cycles (Fig. 3F, two left panels). This trend was not observed among the slow progressors (Fig. 3F, two right panels).

For five out of the seven long‐term survivors, samples from before and after confirmation of progression were available. We noted a marked trend toward increased abundance of M‐MDSCs following progression (Fig. 3G); however, this did not reach statistical significance (*P*_adj_ = 0.0537).

### 3.6 PD‐L1 expression is upregulated on T cells during oleclumab/durvalumab treatment

**Fig. 4:**
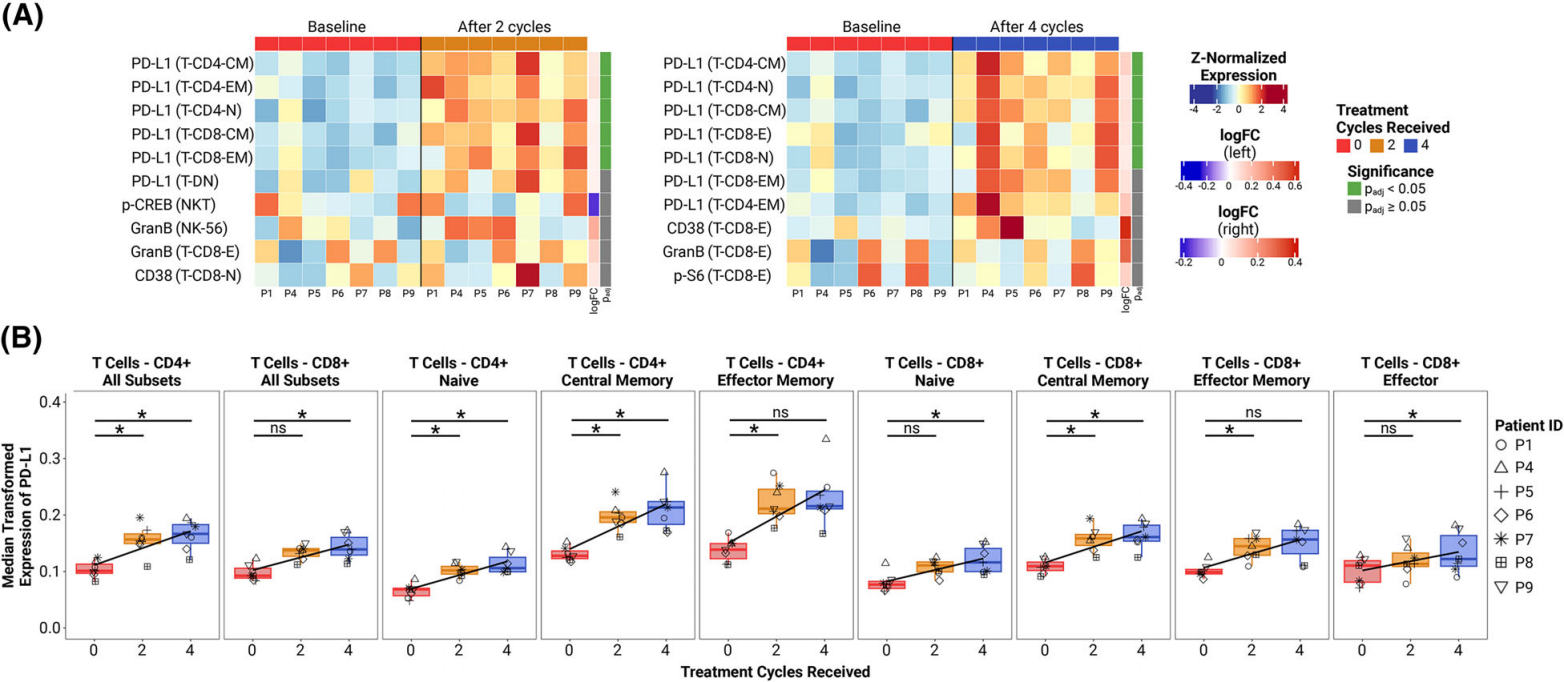
Significant changes in cell state marker expression during treatment. (A) Heatmaps of the most significant differences in the relative state marker expression in long‐term survivor samples taken at baseline (*n* = 7) (left half of the heatmaps) and after either two (*n* = 7) (right half of left heatmap) or four treatment cycles (*n* = 7) (right half of right heatmap). Each row of the heatmap is labeled with the combination of marker (left) and subset (right, in brackets) it represents, sorted from top to bottom based on increasing *P*‐value. (B) Median arcSinh(5)‐transformed expression of PD‐L1 over time for all T‐cell subsets in the long‐term survivor (*n* = 7) samples. Box and whisker plots represent the median (central line), interquartile range (IQR) (box), and minimum and maximum values within 1.5 × IQR out from the IQR (whiskers). *P*‐values were calculated using the “limma” method of the diffcyt R package, which employs linear modeling and empirical Bayes moderated *t*‐tests [29], then corrected for false discovery rate using the Benjamini–Hochberg method. Statistical analyses were performed on paired data. Trendlines were modeled using simple linear regression. ns, not significant; *P_adj_ < 0.05. CM, central memory; E, effector; EM, effector memory; GranB, granzyme B; logFC, log_2_(fold change); Mono, monocytes; N, naïve; NC, non‐classical; NK‐56, CD56^+++^ NK cells; NKT, NKT cells; T‐CD4, CD4^+^ T cells; T‐CD8, CD8^+^ T cells; T‐DN, CD4^−^CD8^−^ (double‐negative) T cells; Tgd, gamma‐delta T cells.

We subsequently performed differential analyses of cell state marker expression levels to investigate inter‐group differences and longitudinal changes in the activation, exhaustion, or terminal differentiation of leukocytes, as well as in the activity of signaling pathways potentially influenced by inhibition of adenosinergic signaling [30]. Differential analyses of state marker expression levels between treatment timepoints and baseline in the long‐term survivors revealed significant upregulation of PD‐L1 expression across nearly all CD4^+^ and CD8^+^ T‐cell subsets (Fig. 4). After two treatment cycles, PD‐L1 expression was significantly increased in all memory (CD45RO^+^) T‐cell subsets and across all CD4^+^ T‐cell subsets, with the CD4^+^ memory T cells showing the highest expression and largest increases (Fig. 4A ‐ left panel; Fig. 4B). Following four treatment cycles, PD‐L1 expression was significantly elevated compared to baseline in all T‐cell subsets except the effector memory (CD27^−^CD45RO^+^CD45RA^−^) T‐cell subsets (Fig. 4A ‐ right panel; Fig. 4B). Notably, the lowest PD‐L1 expression among the T‐cell subsets was observed in the naive (CD27^+^CD45RO^−^CD45RA^+^) T‐cell subsets (Fig. 4B). Interestingly, no significant changes or trends were observed in the expression levels of markers associated with the adenosinergic signaling pathway (CD39, CD73, p‐CREB, and p‐S6) (Tables S4 and S5). Comparisons of cell state marker expression between rapid and slow progressors at all three timepoints, as well as between pre‐ and post‐progression samples, did not reveal any significant differences (Tables S6–S9).

### 3.7 Baseline expression levels of CD73 and IDO1 positively correlate with the duration of progression‐free survival

**Fig. 5:**
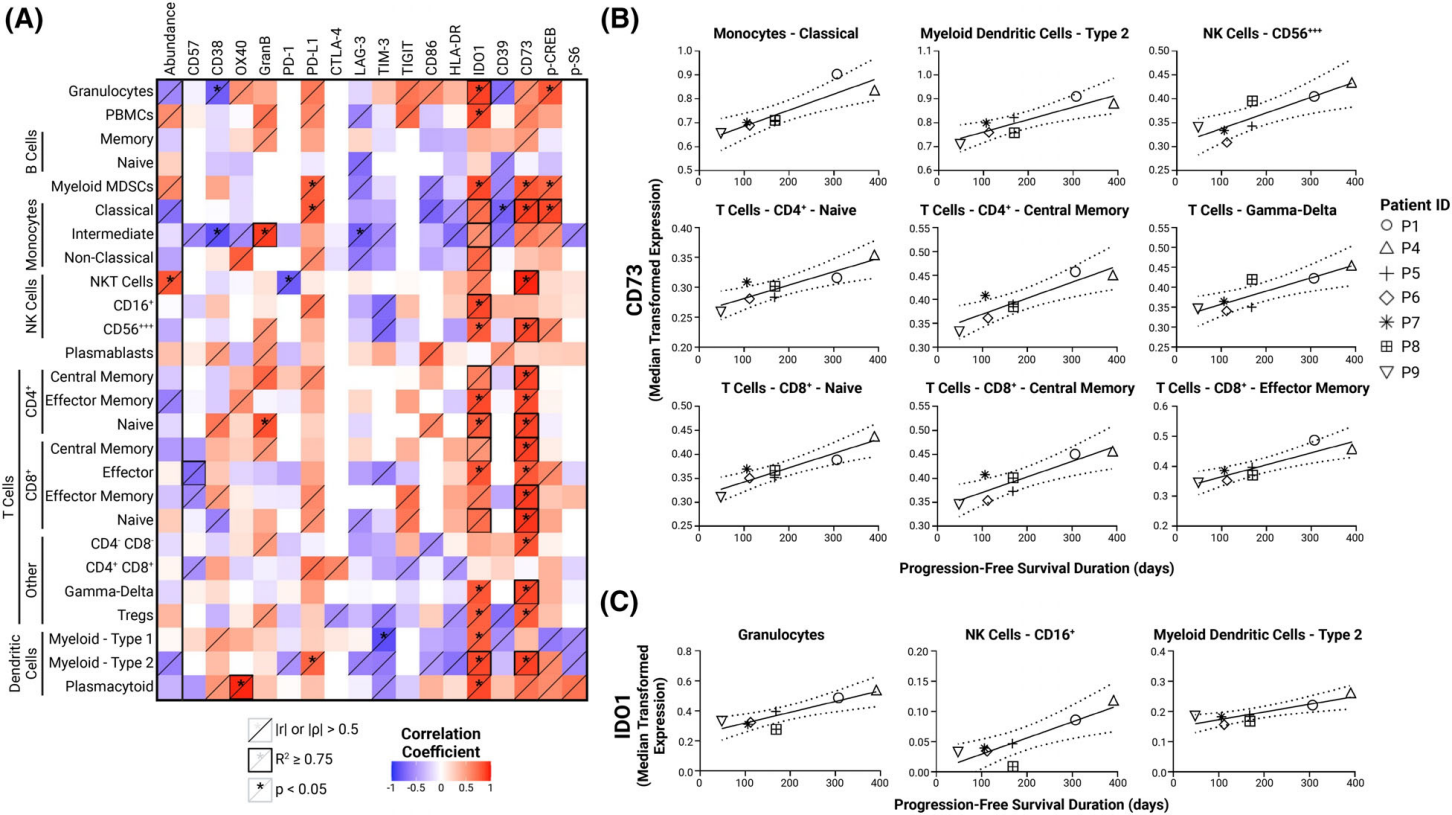
Correlations between progression‐free survival duration and either the relative leukocyte subset abundances or state marker expression levels at baseline for long‐term survivors (*n* = 7). (A) Heatmap showing correlation analysis results (Table S10). Pearson or Spearman correlation analysis was selected based on the normality of the dataset, assessed using the Shapiro–Wilk test. (B) Plots depicting significant correlations between median transformed CD73 expression at baseline and time to progression. Symbols representing each patient are consistent throughout this figure. (C) Plots illustrating most significant correlations between median transformed IDO1 expression at baseline and time to progression. Trendlines in the correlation plots were modeled using simple linear regression. The dotted lines in the correlation plots represent the 95% confidence interval. |*r*|, absolute value of Pearson's correlation coefficient; |ρ|, absolute value of Spearman's correlation coefficient; GranB, granzyme B; PBMCs, peripheral blood mononuclear cells; TCRgd, gamma‐delta T‐cell receptor.

To assess the impact of baseline abundances and phenotypes of peripheral blood leukocytes on PFS duration, we performed correlation analyses. No significant valid correlations were found between baseline subset abundances and PFS duration (Fig. 5A, Table S10). However, analyses of cell state marker expression revealed a strong positive correlation between baseline CD73 expression and PFS duration for most of the subsets comprising the patients' peripheral blood leukocyte repertoire (Fig. 5B). This correlation met the predefined robustness criteria in approximately one third of leukocyte subsets, predominantly encompassing the majority of the T‐cell repertoire. Similarly, a strongly positive correlation with PFS duration was also observed for baseline IDO1 expression in a number of leukocyte subsets, although this correlation was significant in fewer subsets than observed for CD73 expression (Fig. 5C).

## 4 Discussion

In this study, we report the findings from a high‐dimensional single‐cell analysis of blood samples from patients with relapsed EOC undergoing combination oleclumab/durvalumab immunotherapy. To the best of our knowledge, this is the first study to employ single‐cell CyTOF to assess blood‐based biomarkers in patients with ovarian cancer undergoing combination immunotherapy. Through differential and correlative analyses of the abundances and phenotypes of peripheral blood leukocytes, we identified phenotypic signatures potentially indicative of disease progression and treatment efficacy. We show that patients with relapsed EOC who present with lower proportions of PBMCs in their peripheral blood leukocyte pool may derive inferior survival benefit from combined oleclumab/durvalumab treatment than patients with higher proportions of PBMCs. During treatment, we saw an increase in the relative abundance of circulating CD14^+^CD16^−^ myeloid cells in the total patient cohort, along with a decrease in T_CM_ cell proportions specific to rapidly progressing patients. We also observed a strong trend toward an increased relative abundance of circulating M‐MDSCs at progression. Furthermore, we noted a significant increase in PD‐L1 expression on circulating T cells during treatment. Finally, we demonstrate that PFS duration was positively correlated with pre‐treatment expression levels of CD73 and IDO1 on several circulating leukocyte subsets.

In our study cohort, long‐term survivors exhibited significantly higher proportions of total PBMCs at baseline compared to short‐term survivors, which aligns with studies on PD‐1/PD‐L1 checkpoint blockade in lung cancer and melanoma demonstrating significant association between lower baseline neutrophil‐to‐lymphocyte ratios and improved progression‐free and overall survival [31, 32]. Correspondingly, patient P4, who exhibited the highest relative abundance of lymphocytes at baseline among all patients (Fig. 2D), was the sole responder in the trial and demonstrated the longest survival. This data suggest that EOC patients possessing an immune system more effectively primed for adaptive response may derive more consistent benefit from immunotherapy.

We observed a significant expansion of the CD14^+^CD16^−^ myeloid cell compartment over the course of the first four treatment cycles. This expansion was initially driven by an increased abundance of HLA‐DR^+/hi^ classical monocytes, and further sustained by an elevated frequency of HLA‐DR^−/lo^ M‐MDSCs. This indicates a shift of the CD14^+^CD16^−^ myeloid compartment toward a more immunosuppressive phenotype. Therefore, a sudden increase in the proportions of M‐MDSCs in the peripheral blood of EOC patients treated with anti‐CD73/PD‐L1 immunotherapy may serve as a potential biomarker of disease progression. Accordingly, higher HLA‐DR expression on cells within the CD14^+^CD16^−^ myeloid cell compartment has been positively associated with improved response to oleclumab [21] and to immunotherapy targeting the PD‐1/PD‐L1 pathway [33]. Furthermore, cancer immunotherapy trials have consistently demonstrated that elevated MDSC abundance is associated with worse outcome in EOC [34], and various other cancers [35, 36, 37]. Growth factors and proinflammatory cytokines produced by cancer cells chronically stimulate myeloid progenitors, driving their differentiation toward M‐MDSCs. Similarly, tumor‐derived factors can induce the reprogramming of classical monocytes already present in peripheral blood toward M‐MDSCs [38]. This expanded population of CD14^+^CD16^−^ myeloid cells, likely representing precursors to tumor‐associated macrophages, can be recruited to ovarian tumors, resulting in enhanced immunosuppression [34]. We hypothesize that EOC's concurrent utilization of a multitude of immunosuppressive mechanisms allows it to adapt and effectively resist targeted immunotherapy through enhancement of immunosuppression mechanisms not currently affected by treatment. Such adaptation may be addressed by modifications or additions to the existing therapeutic approach. In line with this notion, MDSC‐targeting agents have been preclinically and clinically evaluated in EOC with promising beneficial effect, also in combination with other immunotherapeutic approaches [34, 39].

Our analysis showed a significant decline in the frequencies of T_CM_ cells in the peripheral blood of rapid progressors. Such a decline may have occurred as a consequence of the evolving immunosuppression in the underlying cancer. This hypothesis is supported by the observation that the T_CM_‐cell decline coincides with a substantial increase in peripheral M‐MDSCs in these patients. T_CM_‐cell homeostasis may have been disrupted by the collective inhibition of the activation and differentiation of effector T cells into T_CM_ cells, and the inhibition of the reactivation and proliferation of tumor‐proximal T_CM_ cells through an adaptive increase in adenosine generation. This effect may have been mediated by upregulation or induction of CD39/CD73 expression on tumor‐recruited MDSCs [40], creating what is known as a “purinergic halo” [41]. The role of adenosine in driving the contraction of the T_CM_ subset is further supported by the study of Mastelic‐Gavillet et al. [30], which showed that T_CM_ cells are especially vulnerable to adenosine‐mediated dysfunction due to their high expression of the A2A receptor.

A novel observation of this study was the significant increase of PD‐L1 on circulating T cells over time during administration of an anti‐PD‐L1 ICI. Research on other solid cancers has identified intratumoral PD‐L1^+^CD8^+^ T cells as drivers of T‐cell exhaustion via the PD‐L1/PD‐1 axis, and as promoters of M2 macrophage polarization [42, 43]. Given that PD‐L1 upregulation on T cells occurs following activation, we postulate that the observed longitudinal upregulation of PD‐L1 on circulating T cells in patients with EOC receiving oleclumab/durvalumab is a consequence of rapidly induced and progressively mutual exhaustion of activated T cells.

Interestingly, we did not observe any significant longitudinal changes or inter‐group differences in the expression of markers associated with adenosinergic signaling (CD39, CD73, p‐S6, and p‐CREB). This contradicts previous findings documenting oleclumab‐induced inhibition and internalization of CD73 on CD4^+^ and CD8^+^ T cells [44]. The stability of adenosinergic signaling observed in our study may be attributable to compensatory upregulation of CD73 expression, which possibly counteracted the expected oleclumab‐mediated internalization.

Our study demonstrates a significant positive correlation between baseline CD73 expression and PFS duration across various leukocyte subsets. This contradicts the prevailing view that CD73 expression in cancer generally correlates with poorer treatment outcome [45]. A limited number of studies have investigated CD73 expression in EOC [18, 46], and ours is the first to link baseline CD73 expression with the outcome of CD73‐targeting cancer therapy. Although the positive correlation between baseline CD73 expression and PFS duration was observed for a range of leukocyte subsets, nearly all T‐cell subsets are present in this set and represent a majority of the significant results. Thus, elevated baseline CD73 expression on peripheral blood T cells may serve as a predictive marker of longer PFS in patients with EOC set to undergo anti‐CD73 immunotherapy. We hypothesize that patients in our cohort who demonstrated higher baseline CD73 expression on peripheral blood leukocytes may have had disease more heavily dependent on adenosinergic signaling for immune evasion, potentially enhancing the effectiveness of anti‐CD73 treatment. Nonetheless, this advantage appears to be temporary, as all patients ultimately experienced progression. This development may be attributed to the inherent immunosuppressive adaptability of EOC, as evident by the observed expansion of M‐MDSCs and upregulation of PD‐L1 in T cells.

In addition to the observed positive correlation between CD73 and longer PFS, our observation of a positive correlation between baseline IDO1 expression and longer PFS during EOC immunotherapy is in alignment with previous reports [47, 48]. Although IDO1 is generally viewed as a contributor to immunosuppression, our findings may be explained by its role in upregulating PD‐L1 through the activation of the aryl hydrocarbon receptor by IDO1‐generated tryptophan catabolites, as observed in solid tumors [49] and murine models of EOC [50]. Consequently, higher IDO1 expression in specific cell subsets could lead to increased PD‐L1 expression on the same cells, potentially enhancing their susceptibility to durvalumab. Indeed, consistent with the study by Fujiwara et al. [47], our data also demonstrated a positive correlation between IDO1 and PD‐L1 expression in leukocytes (Table S11). This correlation was significant and robust in subsets that showed the strongest associations between PD‐L1 expression and PFS duration, namely, classical monocytes, M‐MDSCs, and CD141^+^ myeloid dendritic cells. Therefore, IDO1 expression in the peripheral blood myeloid compartment may serve as a potential biomarker for the efficacy of EOC immunotherapy targeting PD‐L1. Despite strong correlation coefficients and significant *P*‐values, the validity of correlations between PD‐L1 and PFS duration was limited by a low goodness‐of‐fit parameter, likely due to the small sample size. Therefore, a larger study is needed to more definitively establish baseline PD‐L1 expression in peripheral blood leukocytes as a predictive biomarker for EOC immunotherapy utilizing anti‐PD‐L1 ICIs.

This study examined sequentially sampled peripheral blood. An advantage of using blood samples, as opposed to tumor tissue biopsies, is that the information provided is not limited to a specific anatomical location. This is particularly valuable in cases of metastatic disease and offers a more comprehensive view of the patient's overall condition. Additionally, blood sampling is more flexible, clinically accessible, cost‐effective, less invasive, and avoids the need for radiological imaging or surgical procedures for sample acquisition. However, the implementation of our 40‐marker panel in the clinical setting is constrained by the high costs and complexity of the CyTOF methodology. Therefore, a more focused set of markers or leukocyte subsets, as suggested in this study, could represent a biomarker panel analyzable via methods already utilized in routine clinical diagnostics, such as flow cytometry or ELISA.

Overall, the small number of patient samples in this study affects the statistical power and limits the generalizability of the results. However, the diversity within the patient population ‐ encompassing a wide range of ages, disease stages, and prior treatments ‐ adds to the robustness and relevance of our observations for a broader spectrum of patients with relapsed EOC undergoing immunotherapy. Furthermore, the use of single‐cell suspension CyTOF allowed us to generate extensive data from the limited sample set. Coupled with high‐dimensional unsupervised clustering algorithms, this approach provided numerous insights and perspectives, strengthening the conclusions of the study.

Another limitation of this study is the absence of control arms comprising patients treated with either oleclumab or durvalumab alone [15]. Such control groups would have provided data integral for the conclusive attribution of the observed differences in leukocyte abundances and states to the effects of a certain immunotherapeutic. In addition, patients had undergone multiple rounds of cancer therapy prior to inclusion, which may have exerted developmental pressure on the tumors and potentially influenced response to oleclumab/durvalumab treatment. Although an expanded trial including single‐agent arms and newly diagnosed EOC patients would help clarify the individual contribution of each compound to treatment outcome, the limited clinical activity of the oleclumab/durvalumab combination in the NSGO‐OV‐UMB1/ENGOT‐OV30 trial [15] makes it difficult and ethically questionable to initiate such a randomized phase II or III trial.

## 5 Conclusions

Our findings offer comprehensive insights into the effects of combination oleclumab/durvalumab immunotherapy on the peripheral blood leukocytes of relapsed EOC patients and elucidate potential immunosuppressive mechanisms employed by EOC to counteract the effects of immunotherapy. We propose blood‐based biomarkers for better patient selection and non‐invasive monitoring of disease progression; however, validation in larger patient cohorts is necessary.

## Conflict of interest

AA has participated on the advisory boards of GlaxoSmithKline and Merck Sharp and Dohme; RDPC is employed by and is a shareholder of Y‐mAbs Pharmaceuticals; LCVT has received personal fees for lectures from Bayer and AstraZeneca, personal fee payments from Eisai for participating on an expert board, and has received a grant related to a clinical trial from AstraZeneca; CEW has received financial support for conference attendance and travel expenses from Beckman Coulter Inc.; EMC is a shareholder of KinN Therapeutics AS; JM has received a honorarium for a lecture from Eisai; KM has received speakers' honoraria and received compensation for travel expenses from GlaxoSmithKline and AstraZeneca, has participated in a trial‐specific safety review committee for Kancera AB, and is a deputy medical director for NSGO‐CTU; MRM has received an institutional study grant and investigational medicinal product from AstraZeneca (no personal grants were received); LB has received honoraria for lectures from GlaxoSmithKline and Merck Sharp and Dohme, has received a research grant from AstraZeneca for a researcher‐initiated trial, and has had leadership roles in Onkologisk Forum between 2018 and 2022 and in the NSGO and NSGO‐CTU since 2021; LT, KK, and KSP report no personal conflicts of interest.

## Author contributions

LT, MRM, and LB contributed to study conceptualization and design. AA, JM, KM, KSP, MRM, and LB contributed to clinical sample collection. RDPC, KM, and KSP contributed to clinical database administration. LT and CEW contributed to data generation. LT contributed to data analysis and statistics. LT, KK, LCVT, EMC, and LB contributed to data interpretation. LT, KK, LCVT, and LB contributed to manuscript writing and editing. All authors contributed to manuscript review. LT, KM, KSP, MRM, and LB contributed to project administration. MRM and LB contributed to funding acquisition and also contributed as guarantors.

### Peer review

The peer review history for this article is available at https://www.webofscience.com/api/gateway/wos/peer‐review/10.1002/1878‐0261.13811.

## Supporting information


**Fig. S1.** Cleanup gating workflow used for the refinement of the raw cytometry by time‐of‐flight (CyTOF) data into intact single cells (singlets).
**Fig. S2.** Sankey diagram of study patient grouping.
**Fig. S3.** Determination of the cutoff of HLA‐DR expression for the separation of classical monocytes and monocytic myeloid‐derived suppressor cells (M‐MDSCs).

**Table S1.** The 40‐marker mass cytometry panel used for leukocyte characterization.
**Table S2.** Detailed data on patient sample leukocyte composition.
**Table S3.** Results of the differential leukocyte abundance analysis between survival duration groups.
**Table S4.** Results of the differential cell state marker expression analysis between treatment cycle 2 and baseline for long‐term survivors.
**Table S5.** Results of the differential cell state marker expression analysis between treatment cycle 4 and baseline for long‐term survivors.
**Table S6.** Results of the differential cell state marker expression analysis between progression‐free survival duration groups at baseline.
**Table S7.** Results of the differential cell state marker expression analysis between progression‐free survival duration groups at treatment cycle 2.
**Table S8.** Results of the differential cell state marker expression analysis between progression‐free survival duration groups at treatment cycle 4.
**Table S9.** Results of the differential cell state marker expression analysis between samples taken after and before disease progression.
**Table S10.** Full correlation testing results.
**Table S11.** Results of correlation testing between IDO1 and PD‐L1 expression at baseline.

**Data S1.** Fixation of Whole Blood Using Phosflow LyseFix.

## Data Availability

The data that support the findings of this study are available from the corresponding author upon reasonable request.
