# Supplementary material for: Peripheral blood leukocyte signatures as biomarkers in relapsed ovarian cancer patients receiving combined anti‐CD73/anti‐PD‐L1 immunotherapy in arm A of the NSGO‐OV‐UMB1/ENGOT‐OV30 trial
Source: Mol Oncol. 2025 Jan 30;19(5):1436–51. doi: 10.1002/1878-0261.13811 (PMC12077279; doi:10.1002/1878-0261.13811)
Supplement: Supplementary file 1 — Fig. S1. Cleanup gating workflow used for the refinement of the raw cytometry by time‐of‐flight (CyTOF) data into intact single cells (singlets). Fig. S2. Sankey diagram of study patient grouping. Fig. S3. Determination of the cutoff of HLA‐DR expression for the separation of classical monocytes and monocytic myeloid‐derived suppressor cells (M‐MDSCs). [file MOL2-19-1436-s001.pdf]

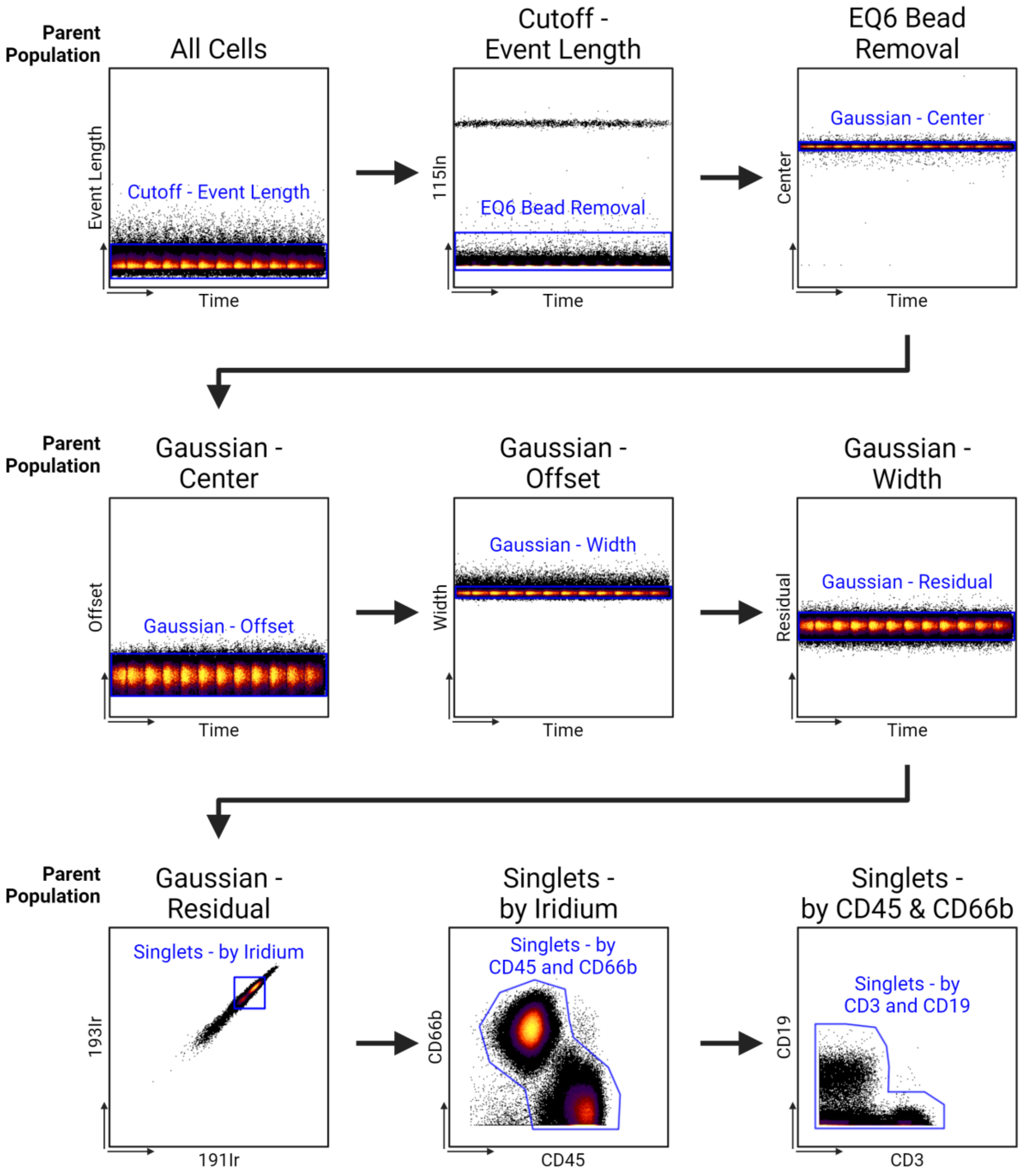

Supplementary Fig. S1 Cleanup gating workflow used for the refinement of the raw cytometry by time-of-flight (CyTOF) data into intact single cells (singlets).

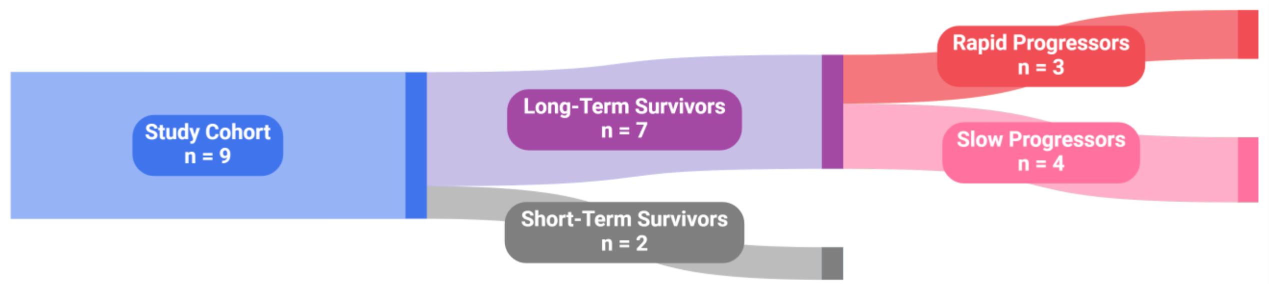

Supplementary Fig. S2 Sankey diagram of study patient grouping. Study patients (n=9) were grouped according to duration of survival and progression-free survival. Patients who survived for up to 16 weeks after the start of treatment were considered short-term survivors (n=2), while patients who survived for longer than 16 weeks after the start of treatment were considered long-term survivors (n=7). Long-term survivors for whom disease progression was confirmed during the first 16 weeks following treatment initiation were categorized as rapid progressors (n=3), while long-term survivors for whom disease progression was confirmed after more than 16 weeks following treatment initiation were categorized as slow progressors (n=4).

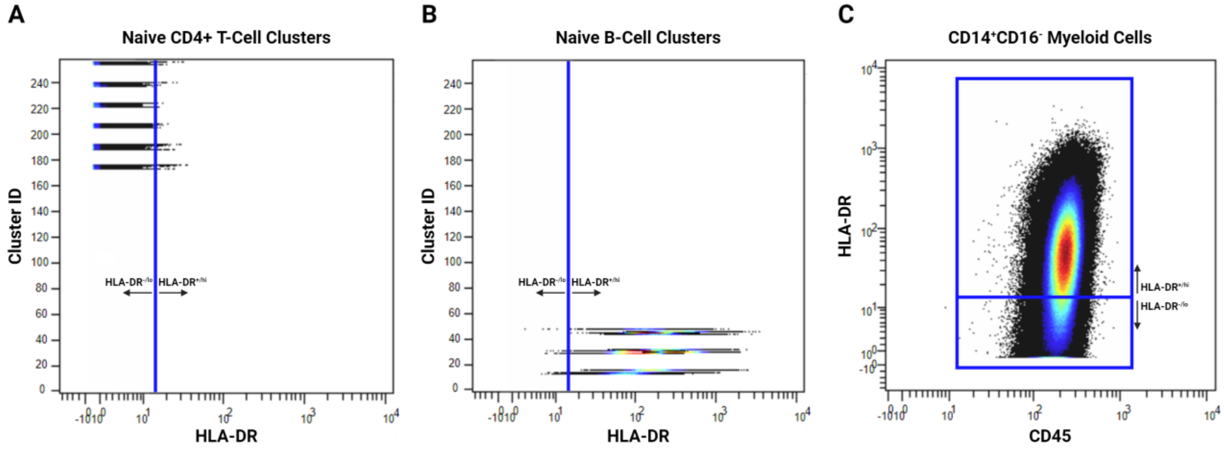

Supplementary Fig. S3 Determination of the cutoff of HLA-DR expression for the separation of classical monocytes and monocytic myeloid-derived suppressor cells (M-MDSCs). (A) The subset of naïve CD4+ T cells was used as a negative control for HLA-DR expression. (B) The subset of naïve B cells was used as a positive control for HLA-DR expression. (C) The cutoff of HLA-DR expression for the separation of the cells of the CD14+CD16- myeloid cell subset into HLA-DR$^{+/hi}$ classical monocytes and HLA-DR$^{-/lo}$ M-MDSCs was set according to the expression cutoffs in the negative and positive control subsets.
